# Brain perivascular macrophages: Recent advances and implications in health and diseases

**DOI:** 10.1111/cns.13263

**Published:** 2019-11-20

**Authors:** Tuo Yang, Ruiming Guo, Feng Zhang

**Affiliations:** ^1^ Department of Neurology Pittsburgh Institute of Brain Disorders and Recovery University of Pittsburgh Pittsburgh Pennsylvania

**Keywords:** multiple sclerosis, neuroinflammation, stroke, vascular dementia, BBB, white matter

## Abstract

Brain perivascular macrophages (PVMs) belong to a distinct population of brain‐resident myeloid cells located within the perivascular space surrounding arterioles and venules. Their characterization depends on the combination of anatomical localization, phagocytic capacity, and molecular markers. Under physiological status, they provide structural and functional support for maintaining brain homeostasis, including facilitation of blood‐brain barrier integrity and lymphatic drainage, and exertion of immune functions such as phagocytosis and antigen presentation. Increasing evidence also implicates their specific roles in diseased brain, ranging from cerebrovascular diseases, Aβ pathologies, infections, and autoimmunity. Collectively, PVMs are key components of the brain‐resident immune system, actively participate in a broad‐spectrum of processes in normal and diseased status. Details of the processes are largely underexplored. Targeting PVMs would lead to new insights and be a promising strategy for a broad array of human diseases.

## 1 INTRODUCTION

Increasing evidence suggests a significant role of brain‐resident immune cells in physiological and pathophysiological settings.^1^ Aside from parenchymal microglia that are extensively studied, border‐associated macrophages (BAMs), including perivascular macrophages (PVMs), meningeal macrophages (MGMs), and choroid plexus macrophages have implicated in variable processes such as development, homeostasis, and diseases.^2^ As their name suggests, PVMs are brain‐resident macrophages located in the perivascular space, also known as Virchow‐Robin space (VRS). Unless peripheral monocytes/macrophages that originate from the mesoderm, PVMs originate from yolk sac and fetal liver embryonically.^2^ Nevertheless, their characterization and their roles in normal and disease status are largely underexplored. In this review, we discuss their characterization focusing on clarifying some confusing anatomical concepts and providing practical suggestions for future research. We also summarize evidence supporting their significant roles in both physiological and pathophysiological states.

## 2 PVM CHARACTERIZATION

**Figure 1:**
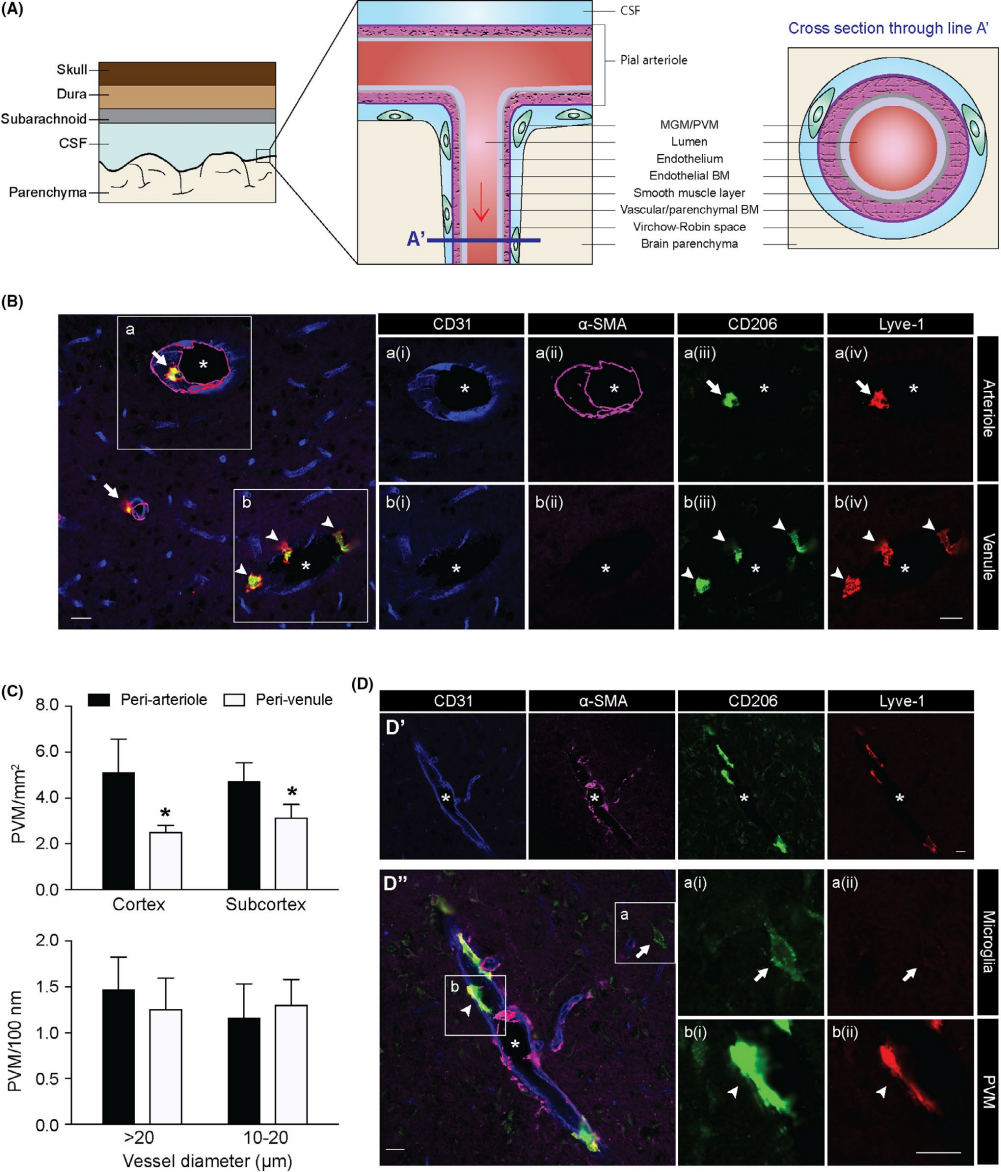
PVM characterization and distribution. A, Anatomical localization of MGMs and PVMs. PVMs are located in the Virchow‐Robin space surrounding pial arterioles and penetrating arterioles. Arrow indicates the direction of blood flow. B, Peri‐arteriole macrophages (arrows) and peri‐venule macrophages (arrowheads). Field a (i‐iv) shows an arteriole with a smooth muscle layer, and field b (i‐iv) shows a venule lacking a smooth muscle layer. *Vessel lumen. Scale bar = 20 μm. C, Perivascular CD206^+^ cell numbers in cortical and subcortical regions of mouse brains (upper panel). Cell numbers normalized to vessel length in vessels of different sizes (lower panel). Data are mean ± SD, n = 3. *P < .05 vs peri‐arteriole by unpaired t test. D, Lyve‐1 is more specific for PVM than CD206. D' shows individual channels of an arteriole. Fields a (i‐ii) and b (i‐ii) in D’’ indicate microglia (arrow, weak CD206, negative for Lyve‐1) and PVM (arrowhead, strong CD206 and Lyve‐1), respectively. *Vessel lumen. Scale bar = 20 μm. CSF, cerebrospinal fluid; MGM, meningeal macrophage; PVM, perivascular macrophage; BM, basement membrane; α‐SMA, α‐smooth muscle actin

It has been about four decades since the discovery of elongated cells in the perivascular space, which possessed autofluorescent large intracellular granules and can uptake exogenous trypan blue and horseradish peroxidase.^3^ Since then, scientists gain more and more knowledge on their identity. Details on the history of PVM discovery and identification are comprehensively reviewed elsewhere.^4^, ^5^ Nowadays, people have come to a consensus that brain PVMs belong to a group of distinct myeloid cells located in the VRS between the vascular basement membrane (BM) on the abluminal side and glial limitans of the brain parenchyma (Figure 1A), and modern PVM characterization relies on a combination of location, function, and molecular markers. Herein we would like to discuss the practical, sometimes confusing issues on these aspects.

### 2.1 Anatomical localization of PVMs

There is controversy about the location of PVMs in relation to BM. Some report that PVMs are positioned outside the BM,^3^, ^6^ while others report the opposite.^2^, ^5^ After careful review of these controversial results, we believe this inconsistency comes from the presence of two layers of BM in the brain vasculature. The inner BM, namely endothelial BM, underlies the endothelium, and the outer BM, namely vascular BM or parenchymal BM, forms the vascular abluminal barrier against glial limitans.^7^, ^8^, ^9^, ^10^ The two BM layers are clearly visible under electron microscopy (EM).^11^ However, immunostaining can hardly illustrate the delicate structure in the current scenario. Both BM layers are positive for laminin^5^ unless different laminin subtypes are stained.^12^ In addition, smooth muscle staining patterns are sometimes irregular 13 (Figure 1B, field a). Therefore, application of EM is much more accurate and trustable, and EM images consistently reveal that PVMs locate between the vascular/parenchymal BM and the glial limitans/brain parenchyma^3^, ^5^, ^6^ (Figure 1A).

Multiple studies have reported the presence of PVMs surrounding musculated vessels, known as arterioles 10‐35 μm in diameter.^4^, ^14^, ^15^ Whether PVMs exist in nonmusculated vessels has not been reported. Although Ookawara et al did mention the possible existence of PVMs surrounding venules, they only identified PVMs surrounding vessels processing continuous or discontinuous smooth muscle layer, excluding the possibility of capillaries.^15^ As an important complement to the field, our group proves the presence of PVMs in both peri‐arteriole space and peri‐venule space (Figure 1B) by showing PVMs surrounding both musculated arterioles and nonmusculated venules. Subsequent cell counting revealed more peri‐arteriole PVMs than peri‐venule PVMs in both cortical and subcortical regions in mouse brain, but no difference if normalized to vessel length (Figure 1C). In addition, we did not observe any difference in PVM distribution with regard to vessel diameter as others reported (Figure 1C).^4^ A reasonable explanation for this discrepancy is that we did not include any pial vessels which are larger in size and harbor much more CD206^+^ cells than intracerebral vessels.^4^

### 2.2 Functional characterization of PVMs

The phagocytotic function of PVMs is utilized for PVM identification. For example, by intravenous (IV) injecting fluorescence labeled dextran, PVMs can be visualized due to their phagocytosis of the fluorescence signals.^6^, ^13^ The physiological significance of PVM phagocytosis will be further discussed under Section 2.2.

**Table 1:** Role of PVMs in neurological diseases

Disease	Model	PVM manipulation	Effect of PVM	References
**Cerebrovascular diseases**				
Ischemic stroke	MCA stroke patients	N/A	Possibly contribute to long‐term poststroke demyelination	60
Ischemic stroke	tMCAO	CLO	Participate in granulocyte recruitment, promote VEGF expression, increase BBB permeability, promote neurological dysfunction	63
Myocardial infarction	SD rats subjected to coronary artery ligation	CLO	Mediate sympathetic activation by releasing proinflammatory cytokines	87
Hypertension	Mice received angiotensin II administration, BPH mice	CLO, bone marrow chimeras	Mediate neurovascular and cognitive dysfunction through oxidative stress	6
High‐fat diet	Mice fed with high‐fat diet	Vegfa^lox/lox^ LysM^Cre+/‐^	Induce VEGF and GLUT1 expression, maintain brain glucose uptake and prevent cognitive dysfunction	64
**Aβ pathologies**				
CAA	TgCRND8 transgenic mice	CLO, chitin (stimulate PVM turnover)	Promote Aβ clearance	16
CAA	J20 transgenic mice	SR‐B1^+/-^ and SR‐B1^−/−^	Promote Aβ clearance, improve neurocognitive function	17
Alzheimer's disease	Aβ topical perfusion, iv administration, Tg2576 transgenic mice	CLO, bone marrow chimeras	Mediate neurovascular dysfunction dependent on CD36 and NOX2	13
**Infectious diseases**				
Bacterial meningitis	Wistar rats received *Streptococcus pneumoniae* intracisternal injection	CLO	Protective, facilitates leukocyte infiltration	73
Viral encephalitis	Breeding pairs of macrophage fas‐induced apoptosis (MAFIA) mice, intranasal injection of VSV	CLO	Detrimental facilitates leukocyte infiltration	18
AIDS and SIVE	Rhesus macaques infected with SIV and HIV human brains	N/A	Express viral DNA, RNA and proteins, reservoir of latent infection	75, 76, 77, 78
**Autoimmunity**				
Multiple sclerosis	Lewis rats injected with MOG	CLO	Promote development of symptoms	83
Multiple sclerosis	Lewis rats injected with MBP or transferred with autoimmune T cells primed by MBP	N/A	Strongly activated and secrete chemokines for monocyte/macrophage recruitment	84
Multiple sclerosis	Recipient mice transferred with autoimmune T cells primed by MOG	Iab^lox/lox^ Cx3cr1^CreERT2^	Not necessary for reactivation of primed autoimmune T cells	85

Perivascular macrophages phagocytosis can also be used to deplete PVMs. Clodronate (CLO) is toxic to PVMs and can kill PVMs once phagocytosed. By intracerebroventricular (ICV) injection of CLO‐containing liposomes, PMV depletion can be achieved. This approach is used for PVM manipulation to study PVM functions.^13^, ^16^, ^17^, ^18^

### 2.3 Markers of PVMs

All leukocytes express CD45 and CD11b, and brain‐resident myeloid cells express fractalkine receptor (Cx3cr1), colony‐stimulating factor 1 receptor (Csf1r), and allograft inflammatory factor 1 (Aif1, also known as Iba‐1).^2^ Compared to microglia, PVMs harbor higher levels of CD45, F4/80, Cx3cr1, and Iba‐1, which can be used to sort PVM with flow cytometry.^2^ PVMs are negative for microglial specific marker purinergic receptor P2RY12, a feature that can be used to distinguish PVMs from microglia.^2^

**Figure 2:**
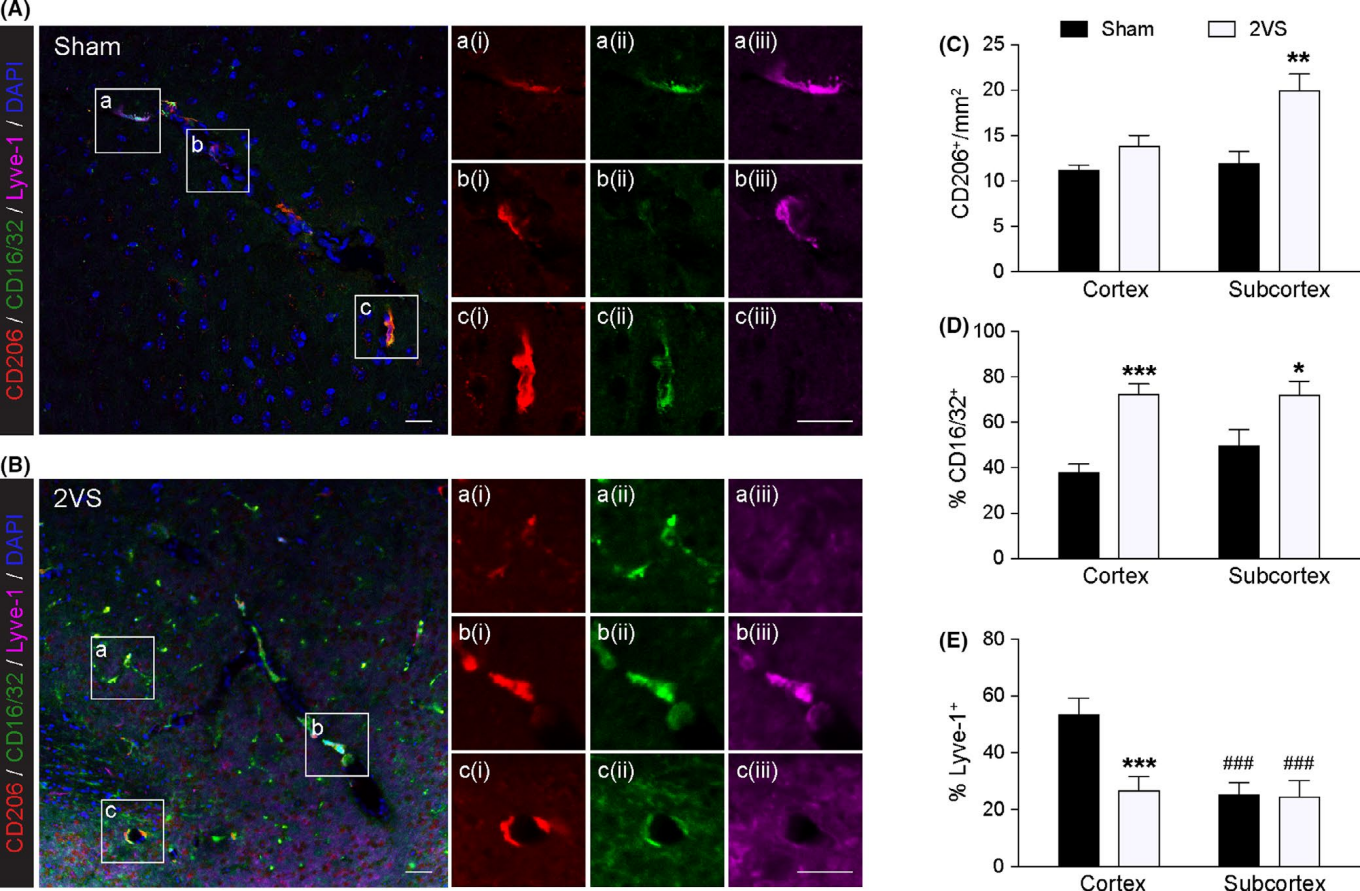
PVM subpopulations in normal and demented brains. Immunostaining of mouse brain slices 8 wk after sham surgery or 2VS. A, PVM subpopulations in Sham brain. Field a (i‐iii) shows a CD206^+^/CD16/32^+^/Lyve‐1^+^ triple positive PVM, b (i‐iii) shows a CD206^+^/CD16/32^−^/Lyve‐1^+^ double positive PVM, and c (i‐iii) shows a CD206^+^/CD16/32^+^/Lyve‐1^−^ double positive PVM of M1 phenotype. Scale bar = 20 μm. B, PVM subpopulations in demented brain. Field a (i‐iii) shows a CD206^+^/CD16/32^+^/Lyve‐1^−^ parenchymal microglia/infiltrating macrophage, field b (i‐iii) shows a CD206^+^/CD16/32^+^/Lyve‐1^+^ triple positive PVM of M1 phenotype, and field c (i‐iii) shows two CD206^+^/CD16/32^+^/Lyve‐1^−^ double positive M1 PVMs. Scale bar = 20 μm. C–E, Total PVM (perivascular CD206^+^ cells) numbers, percentage of CD16/32^+^ PVMs, and percentage of Lyve‐1^+^ PVMs in Sham and 2VS groups. Data are mean ± SD, n = 3. ^*^, ^**^, ^***^ *P* < .05, .01, .001 vs Sham. ^###^ *P* < .001 vs Cortex by 2‐way ANOVA. PVM, perivascular macrophage; 2VS, 2‐vessel‐stenosis.

Conventional PVM markers are CD163, CD206, and lymphatic vessel endothelial hyaluronan receptor‐1 (Lyve‐1). CD163 is a scavenger receptor (SR) protein that recognizes and endocytoses hemoglobin/haptoglobin complexes and involves in antigen presentation. CD163 is highly expressed in PVMs but not in microglia.^19^, ^20^ Another celltype that expresses CD163 is monocyte.^21^ CD206 is a mannose receptor and another widely accepted marker for PVM. CD206 is not present in monocytes, making it more specific to PVMs than CD163.^22^, ^23^ A very small portion of microglia may also express weak CD206 under naïve state (Figure 1D’’, field a), and after brain injury such as stroke and brain trauma, a subpopulation of microglia and infiltrating macrophages transiently express high level of CD206.^24^, ^25^ Lyve‐1 is a receptor for hyaluronan and is expressed on the lymphatic vessels and PVMs, but not monocytes or microglia,^26^, ^27^ making it a more specific PVM marker than CD163 and CD206 (Figure 1D’’, field b). However, Lyve‐1 is not as sensitive as CD206 to identify PVMs (Figure 2A, field c).

In summary, there is no single marker defining PVMs with good sensitivity and specificity by far. Selection of PVM markers is dependent on which assay is used and which cell type is the most crucial to differentiate. For example, CD11b^+^CD45^high^CD206^+^ is a good way for PVM isolation with flow cytometry. In immunostaining, CD163 is good to tell PVMs from microglia but not monocytes. CD206 is good to tell PVM from monocyte, but worse than Lyve‐1 in distinguishing PVM from microglia. A most recent research using mass cytometry followed by single‐cell RNA sequencing revealed CD38 combined with major histocompatibility complex (MHC)‐II to be reliable molecular signatures of BAMs,^28^ but needs further validation. In addition, a combination of anatomical localization and phagocytotic function is more reliable in PVM identification.

### 2.4 Relation of PVMs with MGMs

BAMs consist of PVMs, MGMs, and choroid plexus macrophages.^2^ Same as microglia, BAMs are derived from yolk sac and fetal liver embryonically.^2^ Unlike choroid plexus macrophages which receive bone marrow replenishment constantly after birth, microglia, PVMs, and MGMs are longevity cells throughout the lifespan under normal status.^2^ After depletion, PVMs and MGMs can be replenished from bone marrow monocytes.^6^, ^13^, ^29^

To our knowledge, by far, none of the PVM characterizing approaches discussed above could definitively distinguish PVM from MGM. First, both of them have phagocytotic activities and are visualized after injection of fluorescence labeled dextran.^13^ Second, depletion methods such as CLO liposomes or Cx3cr1 knockout nonselectively deplete both of them,^30^ and repopulation from bone marrow monocytes happens to both of them.^6^, ^13^, ^29^ Third, they share the same set of markers, such as CD206, CD163, Lyve‐1, and MHC‐II.^4^, ^29^ As a result, anatomical localization seems to be the last resort to differentiate them, but only to a limited degree. When focusing on macrophages in VRS surrounding penetrating vessels, one can comfortably say PVM. Problem emerges when it comes to the pia, which holds up pial arterioles and MGMs parallelly. Since MGMs tend to evenly distribute on the pia surface, some MGMs may happen to overlap with pial arterioles while others may not.^4^, ^31^ As for those overlapping with pial arterioles, it is hard to define if they are MGMs or PVMs. Out of this concern, all the data in the present article exclude the pial vessels and only focus on penetrating vessels.

Single‐cell RNA sequencing may be a promising tool to distinguish PVM from MGM, although existing studies fail to do so,^27^, ^32^ as they applied a large scope focusing on whole brain myeloid cells. Narrowing down the scope by focusing only on BAMs may provide useful information.

## 3 PVM FUNCTIONS IN THE NORMAL BRAIN

Outlined below are some of the functions carried out by PVMs in healthy adult brain. Many of them are also implicated in disease settings which will be discussed in detail in Section 3.

### 3.1 BBB integrity

Under physiological conditions, PVMs contribute to BBB integrity. Area postrema is a part of brainstem lacking tight junctions and demonstrates physiologically high BBB permeability. Perivascular macrophages in this region were found to sequester 10‐70 kDa serum proteins from the blood, and along with laminin layer, play a critical role in restricting macromolecules over 10 kDa into the brain.^33^ Similarly, PVMs in peripheral organs restrict vascular permeability, such as mesentery arteries^34^ and cochlear.^35^ Comparable finding is also reported in an in vitro vestibular BBB co‐culture system consisting of endothelial cells (ECs), PVMs, and pericytes.^36^ Interestingly, PVMs may lead to BBB disruption in diseased status. Details are discussed under Section 3.1.

### 3.2 Phagocytosis

The existence of CD163^+^ “perivascular microglia” has long been observed in both rodents 11, 37 and humans.^19^, ^38^ In 1993, Kida et al^39^ identified CD163^+^ “perivascular scavenger cells” in rat cerebral perivascular space, which are distinct from pericytes by containing dense lysosomal bodies under electron microscopy. These cells can phagocytose carbon‐containing Indian ink injected into the caudoputamen from 3 days to 2 weeks after the injection. Subsequently, Mato et al^14^ report that PVMs express type I and type II SRs and uptake multiple exogenous molecules administered via different routes, including acetyl‐low density lipoprotein via IV injection, horseradish peroxidase via IV injection, and ferritin via ICV injection.

Nowadays, the phagocytic ability of PVMs is broadly accepted.^4^ Interestingly, this ability can be restored in PVMs that are repopulated from bone marrow replenishment after experimental PVM depletion. In a bone marrow chimera in which irradiated recipient mice received bone marrow transplantation of green fluorescent protein (GFP)‐labeled monocytes, perivascular GFP^+^ donor cells could be observed as early as 2 weeks after bone marrow transplantation.^40^ In addition, phagocytosis‐dependent labeling of donor PVMs was observed 4 hours after ICV injection of biotin and rhodamine‐labeled dextran (10 kD).^40^

### 3.3 Antigen presentation

Expression of MHC class II is a key feature of antigen presenting cells (APCs).^41^ Back to 1988, MHC‐II‐expressing “perivascular microglia” have been found to present antigen to lymphocytes in an experimental allergic encephalomyelitis (EAE) model.^42^ MHC‐II‐expressing PMVs have also been observed in mouse pineal gland at naïve status,^43^ which are more evident in diseased status, such as mouse retina treated by interferon γ and CD40,^44^ rat brain after transient middle cerebral artery occlusion (MCAO),^45^ mouse EAE brain,^42^ and autopsy from multiple sclerosis (MS) human brain.^22^

### 3.4 Lymphatic clearance

Four lymphatic clearance pathways have been identified in the brain, namely olfactory pathway, meningeal pathway, glymphatic pathway, and intramural perivascular pathway.^46^ Given their close relationship to vessels, PVMs may facilitate the latter two pathways.

First reported by Iliff et al,^47^ the glymphatic pathway involves the “paravascular space,” which drains interstitial fluid and cerebrospinal fluid (CSF) from the para‐arterial space via glial parenchyma to the para‐venous space, finally into the internal cerebral vein. As a low‐resistant drainage pathway, pulsatile paravascular flow generated by the cardiac cycle was observed.^48^ Of note, the so‐called “paravascular space” referred to by Iliff is located between the glial limitans and the vascular BM, which is actually VRS where PVMs reside.

Carare et al^49^ reported the existence of another pathway that involves intracerebral arteries, termed the intramural perivascular drainage (IPAD) pathway. As the term “intramural” suggests, IPAD involves a route within the vessel wall, to be specific, the tunica media which consists of vascular smooth muscle cells (VSMCs). They find that tracers injected into the caudate putamen could gain access into the arterial wall, and travel along the intercellular spaces among VSMCs. PVMs facilitate IPAD clearance by taking up particles ranging from 2 nm to 1 μm.^49^ Interestingly, the continuous flow of IPAD pathway relies on VSMC contraction rather than the arterial pulsation from the heart.^50^ PVMs play an important role in tuning the vascular tone, as ablation of PVMs result in increased regional blood flow, probably through VSMC relaxation.^6^, ^13^ Therefore, PVMs may help control the velocity of IPAD through regulating VSMC constriction and relaxation. Of note, the velocity of IPAD is much slower in aged mice.^51^

In summary, PVMs may facilitate lymphatic drainage by two means. First, as they directly access to lymphatic routes, such as paravascular space and intramural perivascular space, they can phagocytose large particles. Second, PVMs may facilitate IPAD indirectly through regulation of VSMC tone and subsequently regulate IPAD velocity. The exact role of PVMs in lymphatic clearance and their alteration with aging need to be addressed in future research.

Taken together, brain PVMs are located at the brain‐peripheral interface with direct contact to the CSF and the brain parenchyma. They express SRs and exert phagocytosis, acting as immune surveillant and APCs, providing structural and function support to the BBB and lymphatic clearance. These roles are important for brain homeostasis.

## 4 PVM ALTERATIONS IN DISEASED BRAIN

In human brains, increased CD163^+^ perivascular cells were found increased in autopsies from traumatic brain injury, intracerebral bleeding, ischemic stroke, and cerebral hypoxia patients,^52^ indicating that PVMs are active players in disease processes. In this section, we discuss the most extensively studied pathological conditions, where PVMs seem to be able to modify the disease progress (Table 1).

### 4.1 BBB disruption

Several studies described PVM accumulation after BBB injury in various disorders. For example, seizure patients were found to have more CD163^+^ PVMs than controls. And in rats with experimental seizure with BBB leakage, the severity of BBB leakage is positively correlated with CD163^+^ PVM cells.^53^ In a mouse BBB injury model induced by transgenic expression of HIV‐1 Tat_1‐86_ protein, phagocytic PVMs were increased by 5‐fold in caudate/putamen.^54^ In a rat traumatic injury model, CD163^+^ PMVs were increased for at least 4 days after.^55^ In retina, PMV migration to the lesion site is evident after osmotic injury to the blood‐retina barrier.^56^

The role of PVMs in BBB permeability is complicated. It is possible that PVMs facilitate BBB integrity under physiological conditions, while participate in BBB disruption under diseased status.^57^ Some evidence is discussed under Section 2.1 in this regard. Another possibility is that PVM may sense BBB leakage and help to seal the hole, since microglia and microphages were reported to do so.^58^, ^59^ Further studies should focus to solve this dilemma.

### 4.2 Cerebrovascular diseases and risk factors

The detrimental roles of PVMs in cerebrovascular diseases are implicated in several studies. Poststroke humans demonstrate demyelination of the lateral corticospinal track in the spinal cord; MHC‐II^+^ PVMs are present in the lateral corticospinal track, even years after the onset of stroke, and they contain vacuoles filled with lipids, presumably myelin materials, suggesting the phagocytic activity of PVMs may contribute to poststroke demyelination.^60^ PMVs are also reported to mediate oxidative stress in vascular dementia associated with hypertension. Administration of angiotensin II led to oxidative stress, neurovascular dysfunction, and decreased regional blood flow, which were abrogated by PVM depletion with CLO.^6^ Subsequent bone marrow chimeras confirm the critical role of PVM‐derived At1r and NADPH oxidase 2 (NOX2). Translationally, PVM depletion in dementia‐prone hypertensive mice could restore neurovascular and cognitive function.^6^

Based on the above findings and the idea of macrophage polarization, we hypothesized that PVMs may exert proinflammatory polarization (M1). To this end, we examined the conventional M1 marker CD16/32 in demented mice 8 weeks after 2‐vessel‐stenosis (2VS).^61^, ^62^ We here report both CD16/32^+^ and CD16/32^−^ PVMs after sham surgery and 2VS (Figure 2A,B), suggesting the existence of PVM subpopulation. 2VS led to increased parenchymal myeloid cells (Figure 2B), along with increased PVM numbers in subcortex but not cortex (Figure 2B,C), probably responsible for more severe white matter injury than gray matter in dementia. CD16/32^+^ PVMs increased in number and percentage after 2VS, while Lyve‐1^+^ PVMs decreased, suggesting a subpopulation shifting (Figure 2B,D,E). Among CD206, CD16/32, and Lyve‐1, CD206 is the most sensitive, and Lyve‐1 is the most specific, which does not label parenchymal microglia/infiltrating microphages as CD206 and CD16/32 do even after brain injury (Figure 2B, field a). In summary, we report the presence of PVM subpopulations and subpopulation shifting after ischemic injury. Future directions include the origins of PVM increase (from circulating monocytes or in situ proliferation), markers for PVM subpopulations, subpopulation shifting, and related PVM behaviors.

Aside from directly secreting proinflammatory factors and acting as proinflammatory cells, PVMs can also facilitate neuroinflammation indirectly by permeabilizing circulating leukocytes passing through the BBB. In a rat stroke model,^63^ although 1‐hour MCAO followed by 24 hours reperfusion did not significantly change the number of CD163^+^ BAMs, these BAMs upregulated the expression of leukocyte chemo‐attractants. Depleting BAMs with CLO decreased granulocyte infiltration, reduced BBB leakage, and attenuated neurological deficits, without affecting the infarct volumes,^63^ suggesting the role of PVM in facilitating leukocyte infiltration.

Contrarily, the protective role of PVM has also been reported in high‐fat diet (HFD) induced cognitive dysfunction. HFD in mice induced acute reduction of glucose transporter 1 (GLUT1), which was then gradually restored upon prolonged HFD in parallel with compensatory upregulation of vascular endothelial growth factor (VEGF) by CD206^+^ macrophages at the BBB.^64^ Myeloid‐specific deletion of VEGF (VEGFa^lox/lox^ LysM Cre^+/‐^) impaired BBB‐GLUT1 expression, brain glucose uptake, and memory formation in obese mice, as well as exacerbated neuroinflammation and cognitive decline in APP‐PS1 mice. A drawback of this study is that VEGF deletion lacked specificity and occurred in all myeloid cells such as microglia and circulating monocytes.^64^

PVMs also play a role in vascular biology. In developing retina, PVMs could inhibit excessive angiogenesis by inducing EC apoptosis.^65^, ^66^ Interestingly after retinal vein occlusion, monocyte‐derived PVMs accumulated to the lesion site and were protective against EC apoptosis.^67^ In multiple tumors, PVMs were reported to support angiogenesis and maintain tumor microenvironments, partially via secreting microvesicles.^68^, ^69^, ^70^ Although the role of PVMs on vascular biology in cerebrovascular diseases remains underexplored, it is implicated that PMVs are actively engaged in vascular biology in both disease pathogenesis and tissue repair. Future study should address its role specifically in cerebrovascular diseases and related risk factors.

### 4.3 Aβ pathology

PVM's phagocytic function is critical in mitigating Aβ pathology, such as Alzheimer's disease and cerebral amyloid angiopathy (CAA). In one study using TgCRND8 mice (overexpression of human Swedish KM670/671NL and Indianan V717F APP mutations under a hamster prion protein promoter), PVM depletion was associated with increased vascular accumulation of Aβ42 and CAA severity. Moreover, stimulation of PVM turnover with chitin promoted Aβ42 clearance and attenuated CAA severity.^16^ In another study, SR class B type 1 (SR‐B1), an HDL receptor that regulates cholesterol efflux from the peripheral tissues to the liver, was exclusively expressed in brain PVMs. Depleting SR‐B1 in J20 transgenic mice (overexpression of human Swedish KM670/671NL APP mutation) led to accelerated cerebrovascular and parenchymal amyloid plaque formation in the cortex and hippocampus, along with worsened neurocognitive deficits, suggesting a critical role of SR‐B1 and PVMs in Aβ clearance.^17^

Aβ could also lead to blunted cerebrovascular responses.^71^, ^72^ PMVs were reported to participate in Aβ‐induced neurovascular dysfunction through CD36 mediated oxidative stress. Park et al^13^ applied three models of Aβ pathology, Aβ topical perfusion onto brain cortex, Aβ intravenous administration, and Aβ overexpression in Tg2576 mice (mice carrying Swedish mutation of APP). All three models were associated with neurovascular dysfunction as detected with whisker stimulation and acetylcholine superfusion. Additionally, selected deletion of CD36 and NOX2 in PVMs by bone marrow chimera could abolish Aβ‐mediated neurovascular dysfunction, and wild‐type PVMs could re‐establish neurovascular dysfunction in CD36^−/−^ mice.^13^ However, clinical outcome of the mice, such as cognitive function and neurobehavioral performance were not evaluated in this study. Collectively, these findings suggest a complicated, bidirectional roles of PVMs in Aβ pathology. SRs, such as CD36 and SR‐B1, may be crucial in Aβ clearance by PVMs.

### 4.4 CNS infection

PVMs mediate BBB disruption in bacterial and viral infections. In a rat *Streptococcus pneumoniae* meningitis model, depleting PVM/MGM impacted leukocyte influx into the CSF despite increased expression chemo‐attractants and vascular adhesion molecules, without affecting leukocyte trafficking capability. Increased CSF bacterial loads and deteriorated the clinical symptoms were observed,^73^ suggesting a protective role of PVMs. On the other hand, in mouse vesicular stomatitis virus‐induced encephalitis, although PVM/MGM depletion also reduced infiltration of granulocytes, CD4 and CD8 T cells, it decreased morbidity, without affecting viral load,^18^ suggesting a harmful role of PVMs.

PVMs are more extensively studied in encephalitis associated with human immunodeficiency virus (HIV), especially HIV encephalopathy (HIVE), a common complication of acquired immune deficiency syndrome (AIDS). Macrophages are primary targets of HIV infection, and increased number of PVMs in HIVE was observed decades ago.^74^ Using simian immunodeficiency virus (SIV)‐infected rhesus macaque models, multiples studies reported the existence of SIV RNA and proteins in PVMs but not microglia.^75^, ^76^, ^77^ What was more, little viral protein was detected at initial infection, but abundant viral RNA and protein were present in terminal stages with AIDS and SIV encephalopathy (SIVE), suggesting that SIV‐infected PVMs participate in viral production in late stage.^75^ HIV DNA was also detectible in presymptomatic stage from HIV‐infected human PVMs without evidence of productive infection, suggesting PMVs may be reservoirs of latent HIV.^78^

An important issue to be addressed is the origin of these productively infected PMVs. CNS trafficking of monocytes was shown to be associated with HIVE, since CD14^+^CD16^+^ macrophages harboring HIV‐p24 antigen were observed in perivascular spaces, microglial nodules, and white matter regions in HIVE patients.^79^ In agreement with the previous report, by labeling bone marrow monocyte progenitors with BrdU, accumulation of CD163^+^BrdU^+^ PVMs were evident in SIVE lesions, confirming the bone marrow recruitment in SIV infection.^80^ However, 69.9% of the PVMs were present in the CNS before SIVE lesion formation, suggesting the resident PVMs were the primary targets of SIV.^80^ Ki‐67^+^ proliferating PVMs were identifiable in both HIV‐infected human brains and SIV‐infected macaque brains, confirming the proliferation of productively infected PVMs.^81^

In summary, in non‐HIV infections, PVMs mediate BBB disruption and leukocyte infiltration and the outcome may rely on the nature of the pathogen (ie, bacterial vs virus). In HIV/SIV infections, PVMs are the primary target of viruses and can serve as viral reservoirs. Productively infected PVMs come from both monocyte recruitment and in situ PVM proliferation.

### 4.5 Multiple sclerosis

In human MS brain specimens, both CD163^+^ PVMs and MGMs were increased in numbers, especially in the center of acute lesions and the edge of chronic lesions. These cells were HLA‐DR^+^ suggesting a possible role in antigen presentation.^82^ Consistently, in a rat EAE model, increased PVM and MGM numbers and CD163 expression were observed prior to the onset of clinical symptoms and infiltration of blood‐borne leukocytes, while depleting PVMs and MGMs could alleviate clinical symptoms of EAE, suggesting a harmful role of PVMs and MGMs in MS.^83^ This is in line with another rat EAE study, showing strongly activated spinal PVMs expressing increased ICAM‐1, VCAM‐1, MCP‐1, and MIP‐1α, which may explain their promoting EAE process.^84^ High‐dimensional cytometry confirmed that BAMs achieved activation profile characterized by increased expression of CD38 and MHC‐II during peak EAE.^28^ These results indicate that PVMs exert APC function and may actively mediate the immune responses.

The necessity of PVMs in MS was assessed in a most recent study with MHC‐II depletion (Iab^lox/lox^) in selected CNS APCs.^85^ CD4^+^ T cells from 2D2 TCR (or MOG 35‐55 specific TCR) transgenic mice were primed by MOG 35‐55, followed by transferring to recipient mice with selective MHC‐II deletion in different APC populations. EAE was successfully established in recipients lacking MHC‐II in microglia (Iab^lox/lox^ Sall1^CreERT2^) or macrophages (including BAMs, Iab^lox/lox^ Cx3cr1^CreERT2^). However, protective effects were present in recipients lacking MHC‐II in myeloid cells (Iab^lox/lox^ Lyz2^Cre±^) or DCs (Iab^lox/lox^ Itgax^Cre±^), suggesting that it was DC, but not microglia or macrophage or BAM, that was required for the reactivation of autoimmune T cells.^85^ Although this study did not fully agree with the previous ones, it did not exclude the possibility that PVMs may help maintaining the peak autoimmune status. Future studies should address whether PVMs play a role in priming T cells to be autoimmune T cells and the effects of PVMs at different stages of the disease.

## 5 FUTURE DIRECTIONS

Current difficulties of PVM research include lack of specific markers and inability to separate PVMs from MGMs. Application of RNA sequencing, protein sequencing, and mass cytometry may provide more clues in the future. In addition, intramural perivascular space and VRS collapse after death, bringing extra difficulty studying PVMs and lymphatic routes in dead tissue.^86^ Live imaging such as two‐photon and multi‐photon microscopy can be a powerful tool in this regard.

Although much progress has been achieved during past decades, the physiological and pathophysiological functions of PVMs are still poorly understood. Besides mentioned in Section 2 and Section 3, huge amount of questions needs to be answered. For examples, within the brain vasculature, how do PVMs interact with VSMCs and pericytes, how do they help control the blood flow, the lymphatic drainage and BBB permeability to various materials, and if they participate in vascular stress and aging; and outside the vasculature, what signals drive their phagocytosis, trafficking, and activation, what triggers their M1‐M2 skewing and subpopulation shifting, and if there is any interaction between them and brain parenchymal cells. Other features such as regional distribution pattern, renewal, gender difference, and aging remain unexplored. Under most diseased status, it seems that they act as double‐edged swords. Considering the growing body of evidence showing their significant roles in both healthy and diseased status, in‐depth studies on PVMs could lead to new insights in our understanding of CNS biology and human diseases.

## CONFLICT OF INTEREST

The author declare no conflict of interest.
